# Internalizing Mental Disorders and Accelerated Cellular Aging Among Perinatally HIV-Infected Youth in Uganda

**DOI:** 10.3389/fgene.2019.00705

**Published:** 2019-08-02

**Authors:** Allan Kalungi, Jacqueline S. Womersley, Eugene Kinyanda, Moses L. Joloba, Wilber Ssembajjwe, Rebecca N. Nsubuga, Jonathan Levin, Pontiano Kaleebu, Martin Kidd, Soraya Seedat, Sian M. J. Hemmings

**Affiliations:** ^1^Department of Psychiatry, Stellenbosch University, Cape Town, South Africa; ^2^Mental Health Project, MRC/UVRI and LSHTM Uganda Research Unit, Entebbe, Uganda; ^3^Department of Medical Microbiology, Makerere University, Kampala, Uganda; ^4^Department of Psychiatry, College of Health Sciences, Makerere University, Kampala, Uganda; ^5^School of Biomedical Sciences, College of Health Sciences, Makerere University, Kampala, Uganda; ^6^Statistics and Data Science Section, MRC/UVRI and LSHTM Uganda Research Unit, Entebbe, Uganda; ^7^School of Public Health, University of Witwatersrand, Johannesburg, South Africa; ^8^MRC/UVRI and LSHTM Uganda Research Unit, Entebbe, Uganda; ^9^Centre for Statistical Consultation, Department of Statistics and Actuarial Sciences University of Stellenbosch, Cape Town, South Africa

**Keywords:** internalizing mental disorders, relative telomere length, HIV+, perinatally HIV-infected youth, Uganda

## Abstract

**Introduction:** Internalizing mental disorders (IMDs) in HIV+ children and adolescents are associated with impaired quality of life and non-adherence to anti-retroviral treatment. Telomere length is a biomarker of cellular aging, and shorter telomere length has been associated with IMDs. However, the nature of this association has yet to be elucidated.

**Objective:** We determined the longitudinal association between IMDs and relative telomere length (rTL) and the influence of chronic stress among Ugandan perinatally HIV-infected youth (PHIY).

**Methods:** IMDs (depressive disorders, anxiety disorders, and post-traumatic stress disorder) and IMDs were assessed using the locally adapted Child and Adolescent Symptom Inventory-5. In 368 PHIY with any IMD and 368 age- and sex-matched PHIY controls without any psychiatric disorder, rTL was assessed using quantitative polymerase chain reaction. Hierarchical cluster analysis was used to generate the three chronic stress classes (mild, moderate, and severe). *t*-tests were used to assess the difference between baseline and 12 month rTL and the mean difference in rTL between cases and controls both at baseline and at 12 months. Linear regression analysis was used to model the effects of chronic stress on the association between IMDs and rTL, controlling for age and sex.

**Results:** We observed longer rTL among cases of IMDs compared with controls (*p* < 0.001). We also observed a statistically significant reduction in rTL between baseline and 12 months in the combined sample of cases and controls (*p* < 0.001). The same statistical difference was observed when cases and controls were individually analyzed (*p* < 0.001). We found no significant difference in rTL between cases and controls at 12 months (*p* = 0.117). We found no significant influence of chronic stress on the association between IMDs and rTL at both baseline and 12 months.

**Conclusion:** rTL is longer among cases of IMDs compared with age- and sex-matched controls. We observed a significant attrition in rTL over 12 months, which seems to be driven by the presence of any IMDs. There is a need for future longitudinal and experimental studies to understand the mechanisms driving our findings.

## Background

Human immunodeficiency virus/acquired immunodeficiency disease syndrome (HIV/AIDS) is a significant global health burden, with approximately 36.9 million people infected globally (UNAIDS, 2018). Both eastern and southern Africa remain the most affected regions, accounting for 45% of the world’s HIV infections (UNAIDS, 2018). Of the over 2 million HIV-positive (HIV+) children globally, 90% reside in sub-Saharan Africa (UNAIDS, 2010). In Uganda, the country with the fifth-highest HIV prevalence in the region, an HIV prevalence of 0.5% has been reported among children aged 0–14, which corresponds to approximately 95,000 children living with HIV in the country (UPHIA, 2016–2017). The introduction of antiretroviral therapy (ART) has led to improved survival of HIV-infected youth (7–17 years); however, the mental health of these youth has received less attention (Mupambireyi et al., 2014). Perinatally HIV-infected youth (PHIY) are faced with a burden of psychiatric morbidity (Kamau et al., 2012), in addition to delayed motor and cognitive development (Le Doaré et al., 2012; Van Rie et al., 2007). Studies undertaken in both the developed (Europe and the United States) and developing world (sub-Saharan Africa) have documented depression rates of between 12.7% and 40% (Musisi and Kinyanda, 2009; Gadow et al., 2012; Kamau et al., 2012; Mellins et al., 2012; Nachman et al., 2012; Lwidiko et al., 2018; Kim et al., 2014) among PHIY. For anxiety disorders, rates of 9% to 32.2% have been reported among PHIY (Kamau et al., 2012; Mellins et al., 2012; Nachman et al., 2012; Kinyanda et al., 2019). 

IMDs are associated with psychological distress (Musisi and Kinyanda, 2009), impaired quality of life, and non-adherence to ART (Walkup et al., 2009; Malee et al., 2011). In addition, patients with IMDs have higher mortality rates than have the general population (Cuijpers and Smit, 2002; Colton and Manderscheid, 2006; Ahmadi et al., 2011; Druss et al., 2011).

IMDs are characterized by quiet, internal distress (Tandon et al., 2011), in contrast to externalizing disorders, where overtly socially negative or disruptive behavior is displayed (Tandon et al., 2011). IMDs with high levels of negative affectivity include depressive disorders (e.g., dysthymic disorder), anxiety disorders (e.g., generalized anxiety disorder and social anxiety disorder), and obsessive-compulsive disorder (Regier et al., 2013; Turygin et al., 2013). Despite intensive research, the diagnosis of IMDs is still largely based on clinical symptoms, with an absence of biological markers to facilitate diagnosis. This is largely because the pathophysiological mechanisms underlying IMDs, such as depression and anxiety, are still largely unknown. Several studies have investigated the association between telomere length (TL) and IMDs, and shorter TL has been reported in adults with depression (Simon et al., 2006; Verhoeven et al., 2014; Cai et al., 2015) and anxiety disorders (Kananen et al., 2010; Verhoeven et al., 2015).

Telomeres are protein-bound deoxyribonucleic acid (DNA) repeat structures at the ends of chromosomes (Lindqvist et al., 2015), and are important in preventing chromosomes from fusing together during mitosis, thus preventing loss of genetic data (Allsopp et al., 1992; Blackburn et al., 2006). They also regulate cellular replicative capacity (Allsopp et al., 1992; Blackburn et al., 2006). During somatic cell replication, telomeres progressively shorten due to the inability of DNA polymerase enzyme to fully replicate the 3′ end of the DNA strand (Allsopp et al., 1992; Blackburn et al., 2006), a process termed as the “end replication problem” (Watson, 1972). This results in a gradual decline in telomere length (TL) over time. Once a critically short TL is reached, the cell is triggered to enter replicative senescence and subsequently cell death (Allsopp et al., 1992; Blackburn et al., 2006). TL provides a metric of cellular age and accounts for roughly 15% of the variance of age (Epel and Prather, 2018). TL has been reported to shorten in a predictable way with chronological age by roughly 20–40 base pairs per year (Cesare and Reddel, 2010). TL is partially genetically determined, with heritability estimates ranging from 36% to 84% (Aviv, 2012) and is highly variable between individuals (Vasa-Nicotera et al., 2005; Njajou et al., 2007). The current study assessed TL as relative TL (rTL), with rTL being proportional to an individual’s TL (Cawthon, 2009).

Several studies in youth have reported associations between adversity and telomere shortening (Shalev et al., 2013; Theall et al., 2013; Drury et al., 2014; Mitchell et al., 2014). Adversity experienced in youth ranges from exposure to traumatic stressors, such as sexual and physical abuse, to social adversities that relate to family structure, parental mental distress, and socio-economic status (SES). Causal associations between stressful life events and early adversities, such as childhood sexual abuse and major depression, are well documented (Kendler et al., 1999;Fergusson and Mullen, 1999; Kendler et al., 2000), with evidence suggesting molecular signatures of stress overlap with major depression (Cai et al., 2015) Biological processes, such as inflammation and oxidative stress, which have been observed in several psychiatric disorders are also associated with telomere shortening (Wolkowitz et al., 2011a; Wolkowitz et al., 2011b), suggesting that telomere shortening may be related to certain psychiatric endophenotypes.

Depression has been considered a syndrome of accelerated aging (Heuser, 2002). The first study to examine leucocyte TL (LTL) in a group of subjects with either major depression or bipolar disorder and aged-matched controls found shorter LTL among cases compared with healthy controls (Simon et al., 2006). A large longitudinal clinical cohort study found shorter LTL among groups who were currently depressed or had remitted depression compared with healthy controls (Verhoeven et al., 2014). However, there was no statistically significant difference in LTL between the currently depressed and remitted depression groups, suggesting that depression may leave an “indelible marker” on LTL. However, in the currently depressed group, a dose–response relationship was observed, with LTL inversely associated with both severity and duration of depression. This dose–response relationship was further supported by a longitudinal study by Shalev et al. (2014), where persistence of IMDs from 11 to 38 years predicted reduced LTL at 38 years of age in a dose-dependent manner among male participants. It is, however, not possible to rule out that LTL was already reduced at the first episode of depression, indicating that shorter LTL could be a risk factor for depression. Indeed, Gotlib et al. (2015) described shorter LTL as a risk marker for depression, where shorter LTL was observed among girls (aged 10–14 years) at increased risk for depression. High risk for depression was assessed as having a mother with a history of recurrent episodes of depression, while low risk was assessed as having a mother with no current or past Axis I disorder during a girl’s lifetime. However, results across studies have been inconsistent. While several other studies have reported shorter LTL among currently depressed individuals compared with controls (Lung et al., 2007; Hoen et al., 2011; Wikgren et al., 2012; Garcia-Rizo et al., 2013), some studies have failed to find an association (Wolkowitz et al., 2011a; Teyssier et al., 2012; Needham et al., 2015; Schaakxs et al., 2015).

Accelerated aging has also been described in anxiety disorders. Using the same study population as described in Verhoeven et al. (2014), the authors reported shorter LTL among subjects with a diagnosis of current anxiety disorder than among controls (Verhoeven et al., 2015). There was, however, no statistically significant difference in LTL between the remitted anxiety disorder group and controls, suggesting that LTL shortening in anxiety disorders may be more reversible than that associated with depression. Needham et al. (2015) reported an association between shorter LTL and a diagnosis of generalized anxiety disorder and panic disorder among women. Kananen et al. (2010) reported shorter LTL among older anxiety disorder subjects (48–87 years of age) compared with controls, and a study by Okereke et al. (2012) reported a dose–response relationship where severe phobia was associated with shorter LTL.

PTSD has also been considered in the context of accelerated aging (Moreno-Villanueva et al., 2013; Miller and Sadeh, 2014). Shorter LTL has been implicated in PTSD, though the effects were primarily explained by early life stress (O’Donovan et al., 2011). Shorter LTL was reported among combat-deployed soldiers with PTSD, compared with those without PTSD (Zhang et al., 2014). There is a need to understand whether telomere shortening is a direct effect of PTSD, whether the development of PTSD and shortening of telomeres are simultaneous effects of increased stress reactivity (Zhang et al., 2014), or whether telomere shortening is a risk factor for PTSD (Malan et al., 2011).

HIV infection has also been found to be associated with shortened telomeres (Oeseburg et al., 2010; Auld et al., 2016). HIV/AIDS may be viewed as a chronic psychological stressor due to the illness and stigma that are associated with the disease (Varni et al., 2012). Since TL has been found to be a marker for chronic stress (Needham et al., 2015), shorter telomeres are expected in HIV/AIDS subjects as compared with the disease-free population.

We hypothesized that in PHIY in Uganda, attrition in rTL over a 12-month period would be greater in cases of IMDs compared with age- and sex-matched controls without any psychiatric disorder. We further hypothesized that cases would have shorter rTL than controls. We thus aimed to determine the longitudinal association between IMDs and rTL and the influence of chronic stress in this relationship.

## Methods

### Study Design

This case–control study was nested within a Medical Research Council/Department for International Development (MRC/DfID)-funded project that investigated mental health among children and adolescents living with HIV/AIDS in Kampala and Masaka districts of Uganda (CHAKA study), which enrolled 1,339 Ugandan PHIY (7–17 years) of black African ancestry (Kinyanda et al., 2019). All participants with any of the IMDs (368 cases) and an equal number of age- and sex-matched controls were selected from CHAKA (*N* = 736) and included in the present study. Both the baseline and 12-month archived blood sample for each of the included participants was retrieved from which genomic DNA was extracted.

### Study Population

Study participants were recruited from two HIV clinics in urban Kampala [Joint Clinical Research Centre (JCRC) and Nsambya Home Care] and three HIV clinics in rural Masaka [The AIDS Support Organization (TASO), Kitovu Mobile Clinic, and Uganda Cares]. All study participants were on ART.

### Procedures

Consenting PHIY, as well as their caregivers, were interviewed using a structured questionnaire. The questionnaire included, among others, socio-demographic characteristics (sex, study site, age, caregiver level of education, and SES), and modules on depression, post-traumatic stress disorder, and anxiety modules from the DSM-5 referenced Children and Adolescent Symptom Inventory-5 (CASI-5) (Gadow, 2013). The CASI-5 was locally adapted for use in Uganda (Mpango et al., 2017). Trained psychiatric nurses and psychiatric clinical officers administered the CASI-5 at two time points (baseline and 12 months). The CASI-5 lists the symptoms of a wide range of psychiatric disorders including major depressive disorder, generalized anxiety disorder, PTSD, and attention-deficit/hyperactivity disorder, among others. Individual CASI-5 items are rated on a 4-point frequency of occurrence scale ranging from never (0) to very often (3). There are several CASI-5 scoring algorithms; however, in the present study we used symptom count cutoff scores that reflect the prerequisite number of symptoms for a clinical diagnosis. At each study visit, 4 ml of blood was withdrawn from each study participant through venipuncture into an EDTA vacutainer and was stored at −80 °C pending DNA extraction.

### Inclusion and Exclusion Criteria

*Inclusion criteria*: i) HIV-infected outpatients, registered with any of the HIV clinics at any of the study sites; ii) aged between 7 and 17 years at the time of enrolment; iii) conversant in English or Luganda, the language into which the research assessment tools were translated; and iv) able to provide written informed consent(caregivers)/assent(adolescents). Cases were subjects who had any depressive disorder [depression or dysthymia (persistent depressive disorder)] or anxiety disorder. Controls were age-and sex-matched without any psychiatric disorder. Persistent IMDs were baseline cases that remained cases at 12 months, while remitted ones were baseline cases that lost disease status at 12 months

*Exclusion criteria*: i) Seriously ill including being unable to understand study procedures and ii) any other psychiatric disorder other than the ones listed above.

### Ethical Considerations

Both CHAKA and the present study were conducted in compliance with the Code of Ethics of the World Medical Association (Declaration of Helsinki). The CHAKA study obtained ethical and scientific clearance from the Uganda Virus Research Institute (UVRI) Science and Ethical Committee (#GC/127/15/06/459) and the Uganda National Council of Science and Technology (#HS 1601). The present study obtained approval from the Higher Degrees Research & Ethics Committee, School of Biomedical Sciences, College of Health Sciences, Makerere University (#SBS 421) and the Health Research Ethics Committee of Stellenbosch University (#S17/09/179). Caregiver provided informed consent for their children/adolescents to participate in the study and for a blood specimen to be drawn for genetics analyses. Adolescents provided further assent to participate in the study. Study participants who were diagnosed with significant psychiatric problems were referred to mental health units at Entebbe and Masaka government hospitals.

### Measure of Chronic Stress

Chronic stress was measured as social disadvantage and variables that were considered to confer social disadvantage were used to construct a composite index for chronic stress. A composite index of chronic stress was constructed from data collected on the following variables: orphanhood (double orphanhood carried a higher chronic stress score vs. single or not orphaned); food availability (not enough food carried a higher chronic stress score vs. enough food); study site (urban carried a higher chronic stress score than rural); and caregiver level of education (no formal education carried a higher chronic stress scores than primary and primary a higher stress score than secondary, etc.). Hierarchical cluster analysis (HCA) was used to generate the different cutoff points for each chronic stress class.

### Chronic Stress Classes

The chronic stress index ranged from 0 to 3.75, with a normal distribution. A total of three chronic stress classes were generated during HCA, i.e., mild, moderate, and severe. The mild class had a chronic stress score of 0 to 1.375, the moderate class had a score of greater than 1.375 to 2.375, and the severe class had a score of greater than 2.375.

### Analysis of Relative Telomere Length

DNA was extracted from blood collected from each participant, using the QiAmp Mini DNA Extraction Kit (Qiagen GmbH, Germany). Extracted DNA was quantified by 260/280 and 260/230 ultraviolet spectrophotometry on the NanoDrop 1000 spectrophotometer V3.7 (Thermo Fisher Scientific, Wilmington, MA). The DNA was subsequently diluted to 5 ng/µl and amplified using the KAPA SYBR FAST qPCR Master Mix (Merck, Darmstadt, Germany) per Cawthon et al. (2002), with slight modifications. Primers specific for telomeric repeats (T) (Cawthon, 2002) and a stably expressed single copy reference gene (S), the human β-globin (HBG1, 5′-GCTTCTGACACAACTGTGTTCACTAGC-3′; and HBG2, 5′-CACCAACTTCATCCACGTTCACC-3′), were used to amplify telomeric repeats and human β-globin, respectively. For the telomere assay, each reaction included 5 µl of KAPA SYBR FAST qPCR Master Mix (Merck, Darmstadt, Germany); 1.35 and 4.50 µM of forward and reverse primers, respectively; 5 ng of genomic DNA; and water in a 10-µl reaction volume. The human β-globin assay was identical to the telomere assay except that 2.0 µM of each of the forward and reverse primers were used. The reactions for the telomeric repeats and the human β-globin gene were amplified on the same 384-well plates. Each participant’s DNA sample was amplified in triplicate. If the threshold cycle (Ct) values of the triplicates of particular samples differed by more than 0.5, those samples were excluded. From the triplicate Ct values, the means were calculated for each sample and used in subsequent calculations. Amplification was performed on the ABI 7900HT Fast Real-Time PCR system (Applied Biosystems, Foster City, CA) using the following thermal cycling profile: 95° C for 3 min, followed by 40 cycles of 95° C for 3 s and 60° C for 30 s, and a dissociation stage of 95° C for 15 s, 64° C for 15 s, and 95° C for 15 s. A calibrator sample was prepared by pooling equal amounts of DNA from each participant for the construction of a standard curve. The calibrator DNA sample was serially diluted 1.68-fold per dilution, to produce a nine-point standard curve, with DNA amounts ranging from 50 to 0.79 ng/µl. After amplification of the serial dilutions, a linear plot of the Ct versus the log value of the input amount of DNA (standard curve) was constructed using ABI’s SDS v.2.3 software. The efficiency of a reaction was also determined from the standard curve of that reaction. Threshold and baseline values were used as determined by the SDS v.2.3 software. All Ct values were corrected for the PCR efficiency, and interplate calibrations were performed using GenEx software (http://www.gene-quantification.de/datan.html).

A validated qPCR method (Cawthon, 2002) was used to determine relative TLs (rTLs) in all samples. First, the mean telomere repeat copy number (tel, T) was normalized to a reference gene (single copy gene) (scg, S) copy number to control for differences in DNA quantity. The T/S ratio is proportional to the average TL. Thereafter, the factor by which the T/S ratio differs between the experimental sample and the calibrator sample is determined to provide an indication of relative average TL:

$$\begin{array}{l} \text{T/S}=2^{-∆\text{Ct}}\\ \text{Relative average TL(tel)}=2^{-∆∆\text{Ct}}\\ \text{where }∆\text{Ct}=\text{Ct(tel)}-\text{Ct(scg)}. \end{array}$$

A T/S > 1 indicates that the average rTL in the sample is greater than that of the reference sample, and a T/S < 1 indicates that the average rTL in the experimental sample is less than that in the reference sample.

### Power for the Study

We calculated the *post hoc* power for our study based on results from a study by Epel et al. (2004). We used the formula of sample size and power for difference in means in case–control studies. We worked on the assumption that cases (individuals with IMDs) would have higher levels of stress than controls (individuals without IMDs). Epel et al. (2004) found a 15% reduction in mean rTL among cases compared with controls. Given a 1:1 ratio of cases to controls and using a 5% level of significance, with 368 cases and controls, our study was well powered (power greater than 80%) to detect any reduction above 4.75% in mean rTL between cases and controls. For instance, a reduction of 5% in mean rTL between cases and controls provided a power of 83.8%.

### Statistical Methods

Statistical analyses were conducted using Stata 15 (StataCorp, TX, USA).

Socio-demographic characteristics were described between cases and controls. Chi-square tests were used to assess the association between the socio-demographic characteristics and IMDs at baseline (cases vs. controls). SES was generated from a scale of nine household items (car, motorcycle, refrigerator, electricity, bicycle, radio, telephone, cupboard, and flask). Each item was weighted in the respective order, a car carrying a maximum weight of 9 and a flask a minimum weight of 1. A total score of items was generated, whose median cutoff of 13 was used to classify low and high SES. A score less than 13 was classified as low SES, while that greater than 13 was classified as high SES. Our study group (Kinyanda et al., 2011) has previously used household items as a measure of SES in rural settings of Uganda. A *t*-test was used to compare CD4 counts between cases and controls to account for any disparity in HIV disease progression.

Outliers were revealed by box and whisker plots and were all removed from the rTL data. The skewed rTL data became normally distributed after removal of outliers.

The distributions of rTL at baseline and at 12 months and the change in rTL were determined using a standardized normal probability plot (P-P plot) (See **Supplementary Materials**). The difference in rTL distribution at baseline and at 12 months was assessed using *t*-tests. The mean difference in rTL between cases and controls was also assessed using *t*-tests. One-way analysis of variance was used to assess whether there were any statistically significant differences between change in rTL and each of the variables of age, sex, study site, caregiver education level, and child education level.

Linear regression was used to i) assess the relationship between rTL and chronic stress, adjusting for sex and age; ii) model the effect of chronic stress on the association between IMDs and rTL, by comparing models without chronic stress to models with chronic stress; and iii) model the effect of age on the relationship between IMDs and rTL. There were missing data for rTL values at baseline or 12 months or both. For all analyses that needed computation of confidence intervals, we computed 95% confidence intervals; statistical significance was set at a *p*-value less or equal to 0.05, while a *p*-value greater than 0.05 but less than 0.07 was considered a trend towards marginal significance.

## Results

Socio-demographic factors were evenly distributed between cases and controls as shown in **Table 1**.

**Table 1:** Distribution of socio-demographic factors between cases and controls.

Variable (*n*)	Case *n* (%)	Control *n* (%)	95% confidence level
Sex			*p* = 0.111
Male (342)	160 (46.8)	182 (53.2)	
Female (393)	207 (52.7)	186 (52.7)	
Study site			*p* = 0.941
Urban (415)	208 (50.1)	207 (49.9)	
Rural (321)	160 (49.8)	161 (50.2)	
Age			*p* = 0.374
7–11 years (389)	202 (51.9)	187 (48.1)	
12–17 years (307)	149 (48.5)	158 (51.5)	
Education level (caregiver)			*p* = 0.371
No formal education (13)	9 (69.2)	4 (30.8)	
Primary (648)	323 (49.9)	325 (50.1)	
Secondary (35)	35/72 (48.6)	37/72 (51.4)	
SES			*p* = 0.459
Low (332)	171/332 (51.5)	161/332 (48.5)	
High (404)	197/404 (48.8)	207/404 (51.2)	
Mean CD4 count at baseline			
	947.04	944.02	*p* = 0.939

CD4, cluster of differentiation 4; primary, 0–7 years of formal education; secondary, 8–14 years of formal education; low SES, 0–13; high SES, > 13. All numbers that do not add up were due to missing data.

Tests of association between different socio-demographic variables and rTL were run to determine potential confounders (**Table 2**). None of the socio-demographic variables were associated with rTL. Study site, age, and SES were significantly associated with chronic stress (*p* < 0.001, *p* = 0.040, and *p* = 0.015, respectively).

**Table 2:** *p*-values for tests of association between socio-demographic variables and rTL change and chronic stress.

Variable	*p*-value (rTL change)	*p*-value (chronic stress)
Sex	0.298	0.122
Study site	0.235	<0.001
Age	0.553	0.040
Caregiver level of education	0.912	0.587
SES	0.897	0.015
Child level of education	0.611	0.364

SES, socioeconomic status.

### Difference in rTL Between Cases and Controls

rTL was normally distributed at both baseline and 12 months. Mean rTL (95%CI) of the combined sample of cases and controls was 1.148 (1.119–1.176) at baseline and 0.905 (0.879–0.931) at 12 months. For cases, mean rTL (95%CI) was 1.198 (1.157–1.239) at baseline and 0.925 (0.886–0.965) at 12 months; while for controls, mean rTL (95%CI) was 1.097 (1.057–1.137) at baseline and 0.884 (0.851–0.917) at 12 months.

At baseline, we found a statistically significant difference in rTL between cases and controls (*p* < 0.001). However, contrary to what we expected, rTL was longer in cases compared with controls. There was, however, no statistical difference in rTL between cases and controls at 12 months (*p* = 0.117). In addition, the change between baseline and 12-month rTL (rTL change) did not differ statistically between cases and controls (*p* = 0.608) (**Table 3**).

**Table 3:** Difference in rTL between cases and controls.

Time point	Group	Obs	Mean rTL	Std. Dev	95%CI	*p*-value
Baseline	Cases	307	1.198	0.364	1.157–1.239	<0.001
	Controls	306	1.097	0.354	1.057–1.137	
12 months	Cases	278	0.925	0.336	0.886–0.965	0.117
	Controls	274	0.884	0.275	0.851– 0.917	
	Cases	231	−0.256	0.473	0.194–0.317	
rTL change	Controls	234	−0.234	0.416	0.181–0.288	0.608

### Differences Between Baseline and 12-month rTL

In the combined analysis of baseline cases and controls there was significant attrition in rTL between baseline and 12 months (*p* < 0.001). This attrition did not differ by internalizing mental disorder (IMD) status (*p* = 0.608). A further stratified analysis of cases only and controls only yielded similar *p*-values of <0.001 (**Table 4**).

**Table 4:** Differences between baseline and 12 months rTL for baseline cases and controls.

Group	Time point	Obs	Mean TL	Std. Dev	95% conf. interval	*p*-value
Total sample	Baseline	465	1.148	0.366	1.115–1.182	<0.001
	12 months	465	0.903	0.314	0.875–0.932	
Cases	Baseline	231	1.190	0.371	1.142–1.238	<0.001
	12 months	231	0.935	0.348	0.889–0.980	
Controls	Baseline	234	1.107	0.356	1.061–1.153	<0.001
	12 months	234	0.873	0.274	0.837–0.910	

### Association Between Chronic Stress and rTL

We observed a trend towards statistical significance between chronic stress and baseline rTL (*p* = 0.067). Severe stress was significantly associated with longer rTL (*p* = 0.028) (**Table 5**). However, chronic stress was not significantly associated with either 12-month rTL or a change in rTL (*p* = 0.147 and *p* = 0.455, respectively) (**Table 5**).

**Table 5:** Assessing association between chronic stress and rTL, adjusted for age and sex.

Time point	Chronic stress class	Coefficient	*p* > |*t*|	95% conf. interval	*p*-value
	Mild	Reference			
Baseline	Moderate	0.013	0.717	−0.055 to 0.080	0.067
	Severe	0.091	0.028	0.010 to 0.173	
	Mild	Reference			
12 month	Moderate	−0.031	0.288	−0.089 to 0.026	0.147
	Severe	0.034	0.334	−0.035 to 0.104	
	Mild	Reference			
rTL change	Moderate	0.058	0.214	−0.034 to 0.149	0.451
	Severe	0.037	0.507	−0.073 to 0.147	

Reference, reference chronic stress class during regression analysis.

### Association Between Chronic Stress and IMDs

We found a trend toward statistical significance between chronic stress and IMDs (**Table 6**).

**Table 6:** Association between chronic stress and internalizing mental disorders.

Chronic stress class	Cases (*n*)	Controls (*n*)	Total	*p*-value
Mild	120	144	264	0.065
Moderate	178	147	325	
Severe	70	77	147	
Total	368	368	736	

### The rTL and IMDs After 12 Months

We found no significant difference in baseline rTL between cases of IMDs that persisted compared to those that remitted after 12 months (*p* = 0.235). We also found no statistically significant association between 12-month rTL and 12-month IMD status (*p* = 0.090), as well as no association between disease severity and rTL at baseline (*p* = 0.238) and 12 months (*p* = 0.264).

#### Effect of Chronic Stress on the Association Between IMDs and rTL

We found no significant influence of chronic stress on the association between IMDs and rTL both at baseline and at 12 months (**Table 7**).

**Table 7:** Effect of chronic stress on the association between IMDs and rTL.

Baseline	Disease status	Coefficient	*p* > |*t*|	95% conf. interval	*p*-value	*R*-squared
Without CS	Controls	Reference			<0.001	
	Cases	0.101	0.001	0.044 to 0.15834		0.020
With CS	Controls	Reference				
	Cases	0.101	0.001	0.044 to 0.15796		
	Mild	Reference				
	Moderate	0.017	0.612	−0.048 to 0.081	<0.001	0.028
	Severe	0.088	0.028	0.010 to 0.167		
12 months	Disease status	Coefficient	*p* > |*t*|	95% conf. interval	*p*-value	*R*-squared
Without CS	Controls	Reference				
	Cases	0.041	0.117	−0.010 to 0.092	0.117	0.005
With CS	Controls	Reference			0.128	0.011
	Cases	0.040	0.128	0.012 to 0.091		
	Mild	Reference				
	Moderate	0.030	0.322	−0.087 to 0.029		
	Severe	0.035	0.316	0.034 to 0.105		

CS, chronic stress; reference, reference disease status/chronic stress class during regression analysis.

### Effect of Age on the Relationship Between IMDs and rTL

On stratifying our analyses for age [children (7–11 years) and adolescents (12–17 years)], we observed no statistically significant differences by age group for IMDs and rTL compared with those that were observed with both age categories combined (**Table 8**).

**Table 8:** Effect of age on the association between IMDs and rTL.

Baseline					
Category	IMDs status	Coefficient	*p* > |*t*|	95% conf. interval	*p*-value
	Controls	Reference			
Total sample	Cases	0.101	0.001	0.044 to 0.158	<0.001
	Controls	Reference			
Children	Cases	0.094	0.025	0.012 to 0.175	0.025
	Controls	Reference			
Adolescents	Cases	0.110	0.007	0.031 to 0.190	0.007
12 months					
Category	IMDs status	Coefficient	*p* > |*t*|	95% conf. interval	*p*-value
	Controls	Reference			
Total sample	Cases	0.041	0.117	−0.010 to 0.092	0.117
w	Controls	Reference			
Children	Cases	0.048	0.181	−0.022 to 0.118	0.181
	Controls	Reference			
Adolescents	Cases	0.033	0.395	−0.043 to 0.109	0.395
rTL change					
Category	IMDs status	Coefficient	*p* > |*t*|	95% conf. interval	*p*-value
	Controls	Reference			
Total sample	Cases	0.021	0.608	−0.060 to 0.102	0.601
	Controls	Reference			
Children	Cases	0.017	0.768	−0.098 to 0.133	0.768
	Controls	Reference			
Adolescents	Cases	0.027	0.639	−0.086 to 0.140	0.639

Children, 7–11 years; adolescents, 12–17 years; reference, reference IMDs status during regression analysis.

## Discussion

In this study, we investigated the association between chronic stress and rTL among PHIY cases with IMDs and age- and sex-matched controls in Uganda. To our knowledge, this is the first sub-Saharan African study to investigate the association between chronic stress with rTL and IMDs among PHIY.

Several studies have determined the association between TL and different internalizing psychopathologies. Shorter TL have been reported among cases of depression compared with controls (Garcia-Rizo et al., 2013; Shalev et al., 2014; Verhoeven et al., 2014), while others have failed to find significant associations (Wolkowitz et al., 2011a; Teyssier et al., 2012; Simon et al., 2015). Shorter TL has also been implicated in both anxiety disorders (Kananen et al., 2010; Verhoeven et al., 2015) and PTSD (O’Donovan et al., 2011; Zhang et al., 2014) and has been reported to confer risk for PTSD (Malan et al., 2011). Due to these reported associations of shorter TL in the different internalizing psychopathologies, we hypothesized that rTL would be shorter among cases of IMDs than controls in our study participants. Contrary to our hypothesis, we observed longer rTL among cases of IMDs compared with their controls (*p* < 0.001). Longer rTL among IMDs could be due to elevated telomerase levels. TL is maintained by a telomerase enzyme component known as telomerase RNA component (TERC) and a reverse transcriptase enzyme known as the telomerase reverse transcriptase (TERT) (Wang and Meier, 2004; Blackburn et al., 2006). Wolkowitz et al. (2012) indeed reported elevated telomerase levels among people with depression than among healthy matched controls at baseline. After 8 weeks of treatment with selective serotonin re-uptake inhibitors, they found that telomerase levels became even more elevated as depression remitted. It has been speculated that elevated telomerase levels are a compensatory effort towards excessive loss of telomeres (Damjanovic et al., 2007; Lin et al., 2012).

We also observed a statistically significant reduction in rTL between baseline and 12 months in a combined sample of cases and controls (*p* < 0.001). A statistical difference was also observed when cases and controls were individually analyzed (*p* < 0.001). This difference was expected since TL generally decreases over the life span (Muezzinler et al., 2013). We found no significant difference in rTL between cases and controls at 12 months (*p* = 0.117). Since cases had significantly longer rTL than controls at baseline (*p* < 0.001), the lack of a significant difference at 12 months indicates greater rTL attrition among cases compared with controls. This is an interesting observation that points to the notion that IMDs are possibly driving accelerated cellular aging (rTL attrition). Indeed, telomere shortening has been reported to be strongly influenced by chronic stress exposure (Ridout et al., 2015), and suffering from a chronic disease, such as heart disease (Haycock et al., 2014) and diabetes (Zhao et al., 2013), has been conceptualized as a prolonged stress exposure that could explain their association with TL. IMDs have been reported as chronic stressors (McEwen, 2003) with chronic biological adaptations that result in long-term biological damage that could potentially explain rTL attrition due to IMDs. IMDs could also be leading to rTL attrition through inflammatory pathways. Depression has been reported to prime larger cytokine responses to stressors (Kiecolt-Glaser et al., 2015). Increased systemic inflammation has been associated with decreased TL among a prospective cohort of workers exposed to high level of fine particulate matter (Wong et al., 2014), while interventions that attenuate inflammatory processes in fear- and anxiety-based disorders have been thought to be effective in mitigating the symptoms of anxiety disorders (Michopoulos et al., 2017).

If IMDs were driving rTL attrition, we would expect significant reduction in rTL among cases with no corresponding significant reduction among controls. Intriguingly, we observed significant reduction in rTL in both groups (*p* < 0.001). This is possibly due to general longitudinal reduction in rTL. However, study subjects were only followed up for 12 months, and a longer follow-up period may be required to see a true difference in rTL attrition between cases and controls. It needs to be borne in mind that other factors may be responsible for either the overall greater reduction in rTL over 12 months or the greater rTL reduction among cases than controls. For example, participants were all on ART, with the type of ART regimen not accounted for in the analysis. Also, factors known to affect rTL, such as diet (Shiels et al., 2011) and frequency of physical exercise (Cherkas et al., 2008) were not accounted for. In addition, effects on rTL may have been determined even before birth from maternal stress, or through direct transmission of maternal rTL.

Although previous studies among children have found associations between TL and socio-demographic variables, such as caregiver level of education (Needham et al., 2012), parental SES (Needham et al., 2012; Mitchell et al., 2014), sex (Drury et al., 2014), and living environments (Theall et al., 2013), we found no association between any baseline socio-demographic variables and rTL change in the present study. This discrepancy could be due to cultural context, as previous studies were carried out in developed world settings that differ from the African low-income setting of this study. For example, stress due to orphanhood in the Ugandan context may be experienced differently, as there is a strong extended family system in Uganda where orphans tend to be taken care of by their uncles or aunts, unlike in the developed world where orphans are often institutionalized. The latter has been associated with shorter TL (Drury et al., 2012). More studies are needed to understand factors that affect TL in the sub-Saharan African context.

We found no association between rTL change and persistence or remission of IMDs. This further suggests that rTL does not drive IMDs, but rather IMDs may be driving accelerated cellular aging. Higher mortality rates have been reported among patients with IMDs compared with the general population, and the mortality is mainly due to the same age-related diseases as the general population, such as cancer, and heart, and cerebrovascular disease. For example, a study by Colton and Manderscheid (2006) reported that clients with a diagnosis of major mental illness died 1 to 10 years earlier than did clients with no major mental illness. Another study reported that persons with mental disorders died an average of 8.2 years younger than did the rest of the population and that presence of a mental illness was associated with a hazard ratio of 2 over a 17-year study period (Druss et al., 2011), supporting the mediating role of IMDs in accelerated cellular aging.

Psychological stress (both perceived stress and chronicity of stress) has been significantly associated with lower telomerase activity and shorter TL (Epel et al., 2004). We investigated the association between chronic stress and rTL in our sample. We observed a marginally significant association between chronic stress and rTL (*p* = 0.067). However, contrary to expectation, severe chronic stress was associated with longer rTL (*p* = 0.028) (**Table 5**). Longer rTL was also associated with IMD caseness. Thus, if increased stress (chronic) is an acquired vulnerability factor for IMDs, then it stands to reason that severe chronic stress would be associated with longer rTL, an association that we indeed observed. Further, since IMDs are associated with impaired quality of life and negative clinical and behavioral outcomes among PHIY and poor adherence to ART (Malee et al., 2011; Walkup et al., 2009), we expected significantly lower CD4 counts among cases than controls. However, we found no significant difference in mean CD4 count between cases and controls (*p* = 0.939). We did not investigate other virologic markers of HIV disease severity, such as viral load. However, all study participants were on ART, and thus no difference would be expected if adherence to treatment was similar between cases and controls.

We observed an association between chronic stress and study site and SES respectively. Living in urban areas and having a high SES were associated with more chronic stress than living in rural areas and having a low SES. The association of both urban location and high SES with increased chronic stress may be due to a correlation between the two variables, as participants in urban areas are often of higher SES as compared with their rural counterparts. The association of urban location with increased chronic stress could be due to ecological factors and pressures that are associated with urban life as compared with rural life. We also observed an association between age and chronic stress. Adolescents (12–17 years) were more stressed than children (7–11 years) and this could be due to the fact that adolescents were aware of their HIV status and the stress could be associated with the burden of being HIV+ and stigma among these study participants (Knizek et al., 2017).

Lastly, since severe chronic stress is associated with longer rTL, we expected severe chronic stress to lower the *p*-value of the regression for the association between IMDs abd rTL, an interaction we did not observe. We think that this could due to duration of chronic stress. Although the chronic stress variables used in the present study are known stressors in this population, the duration of the stressor was not assessed for.

## Limitations and Recommendations

We defined IMDs as having any depressive disorder or anxiety disorder or PTSD. The inclusion of PTSD is contentious as the disorder has recently been delineated from IMDs in the DSM-5 and may have skewed our findings. We recommend that future studies undertake a comparative analysis of the different disorders that make up the IMD spectrum to elucidate the independent contribution of each particular disorder.

We did not investigate factors that are known to affect rTL, such as frequency of physical activity, medication, diet, and presence of other comorbid diseases. Also, much as CD4 counts did not significantly differ between cases and controls, the ART regimen for each study participant was not accounted for in the analysis. Future studies should endeavor to consider these factors.

Both the duration and severity of IMDs have been shown to affect rTL. We did not assess the duration of IMDs. However, we think that this may not have greatly affected our findings because disease severity was not significantly associated with rTL in the present study. Future studies should, however, account for the duration of IMDs.

We suggest that the longer rTL observed among cases is due to elevated telomerase activity/levels. However, we did not investigate telomerase activity/levels between cases and controls. Future studies should investigate this possibility. Also, certain genes, such as the telomerase reverse transcriptase and telomerase RNA component, have been reported to influence TL biology. The role of polymorphisms in these genes influencing rTL needs to be investigated, and future studies should endeavor to address this.

Chronic stress was measured using a number of context-specific indicators because there is no locally adapted tool for assessing chronic stress in this setting. While this may be a limitation and may limit generalizability to other settings, the variables used to generate the chronic stress index are known stressors in this population. Validation of the chronic stress index tool will be required in future studies in Uganda.

## Conclusions

RTL was longer in cases with IMDs compared with age- and sex-matched controls.

We observed significant attrition in rTL over 12 months. This rTL attrition seems to be driven by the presence of any IMDs, indicating that IMDs could be driving accelerated rTL attrition. Mechanisms that either directly influence rTL or alleviate the effects of IMDs on rTL attrition could explain our study findings, and longitudinal and experimental studies are needed to fully elucidate underlying mechanisms. 

## Ethics Approval and Consent to Participate

The study obtained ethics approval from the Health Research Committee of Stellenbosch University (#S17/09/179) and the higher Degrees Research & Ethics Committee of the School of Biomedical Sciences, College of Health Sciences, Makerere University (#SBS 421). The parent study (CHAKA) obtained ethics approval from the Uganda Virus Research Institute’s Science and Ethical Committee (#GC/127/15/06/459) and the Uganda National Council of Science and Technology (#HS 1601). All caregivers provided informed consent for their children/adolescents to participate in the study and for a blood specimen to be withdrawn from them (child/adolescent) for rTL and other genetics analyses. Adolescents further provided informed assent to participate in the study.

## Consent for Publication

No details, images, or videos relating to any of the study participants are included in this manuscript.

## Ethics Statement

The study obtained ethics approval from the Health Research Committee of Stellenbosch University (# S17/09/179) and the Higher Degrees Research & Ethics Committee, School of Biomedical Sciences, College of Health Sciences, Makerere University (# SBS 421). The parent study (CHAKA) obtained ethics approval from the Uganda Virus Research Institute (UVRI) Science and Ethical Committee (# GC/127/15/06/459) and the Uganda National Council of Science and Technology (# HS 1601). All study participants provided written informed consent/assent to participate in the study and for a blood specimen to be withdrawn from them for the rTL and other genetics analyses in accordance with the Declaration of Helsinki.

## Author Contributions

Concept was provided by AK, SMJH, EK, and SS. Data collection was done by AK, EK, SMJH, JSW, and SS. Data analysis was done by WS, AK, RNN, SMJH, JSW, SS, MK, and JL. First draft was done by AK, SMJH, JSW, WS, EK, SS, MLJ, RNN, PK, MK, and JL. Final revision was done by AK, SMJH, JSW, EK, SS, WS, MLJ, RNN, PK, MK, and JL. All authors read and approved the final manuscript.

## Funding

The study was funded by Medical Research Council/Department for International Development—African Leadership Award to Prof. Eugene Kinyanda (grant number MR/L004623/1), the Alliance for Global Health and Science of the Center for Emerging and Neglected Diseases (grant number 50288/N7145), the South African Research Chairs Initiative in Posttraumatic Stress Disorder, funded by the Department of Science and Technology, and the National Research Foundation of South Africa.

## Conflict of Interest Statement

The authors declare that the research was conducted in the absence of any commercial or financial relationships that could be construed as a potential conflict of interest.

## Abbreviations

µL, microliter; ART, anti-retroviral therapy; CASI-5, Child and Adolescent Symptom Inventory—edition 5; CD4, cluster of differentiation 4; CIs, confidence intervals; Ct, threshold cycle; DNA, deoxyribonucleic acid; DSM-5, *Diagnostic Statistical Manual for Mental Disorders*—edition 5; HBG, human β-globin gene; HCA, hierarchical cluster analysis; HIV/AIDS, human immunodeficiency virus/acquired immunodeficiency disease syndrome; HIV+, HIV positive; IMD, internalizing mental disorder; JCRC, Joint Clinical Research Centre; LTL, leucocyte telomere length; MRC/DfID, Medical Research Council/Department for International Development; ng, nanogram; PTSD, post-traumatic stress disorder; qPCR, quantitative polymerase chain reaction; rTL, relative telomere length; s, second; scg, single copy gene; TASO, The AIDS Support Organization; TERC, telomerase RNA complex; TERT, telomerase reverse transcriptase; TL, telomere length; UNAIDS, The Joint United Nations Programme on HIV and AIDS; UVRI, Uganda Virus Research institute.
