# Supplementary figures and images for: Internalizing Mental Disorders and Accelerated Cellular Aging Among Perinatally HIV-Infected Youth in Uganda
Source: Front Genet. 2019 Aug 2;10:705. doi: 10.3389/fgene.2019.00705 (PMC6688656; doi:10.3389/fgene.2019.00705)

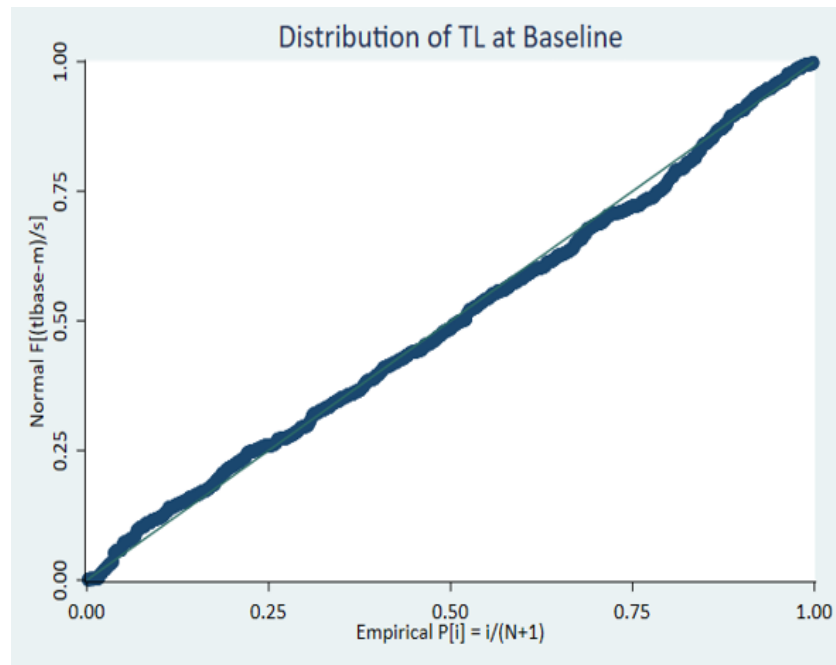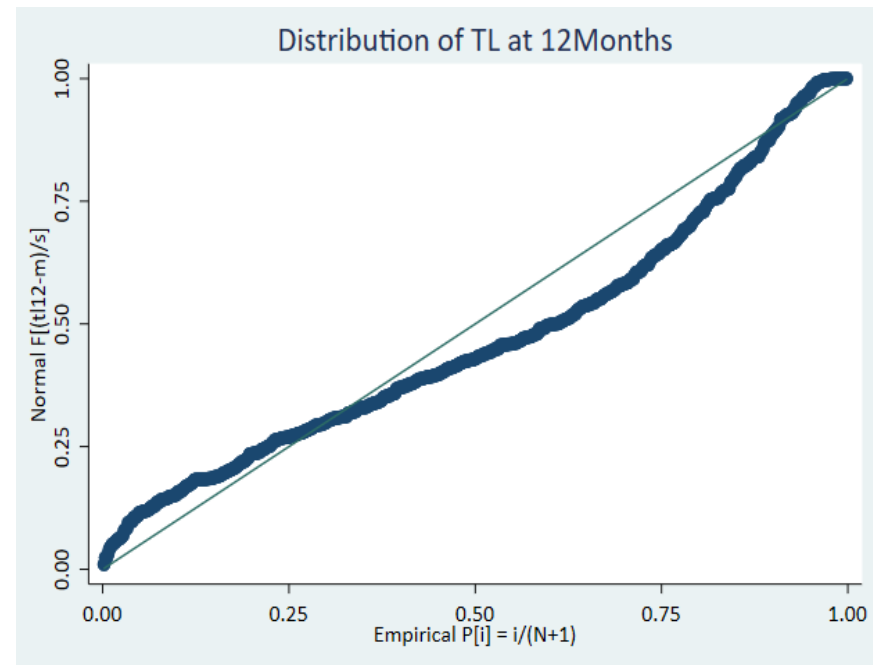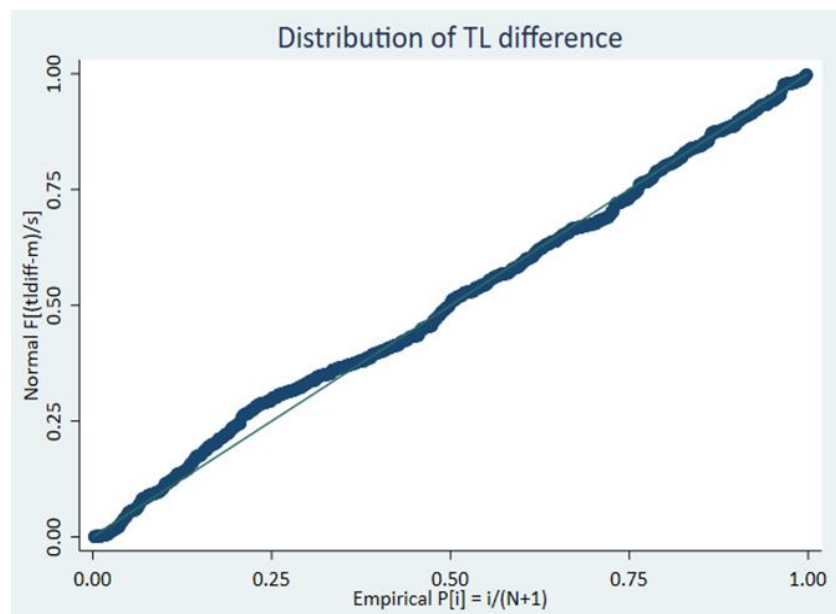

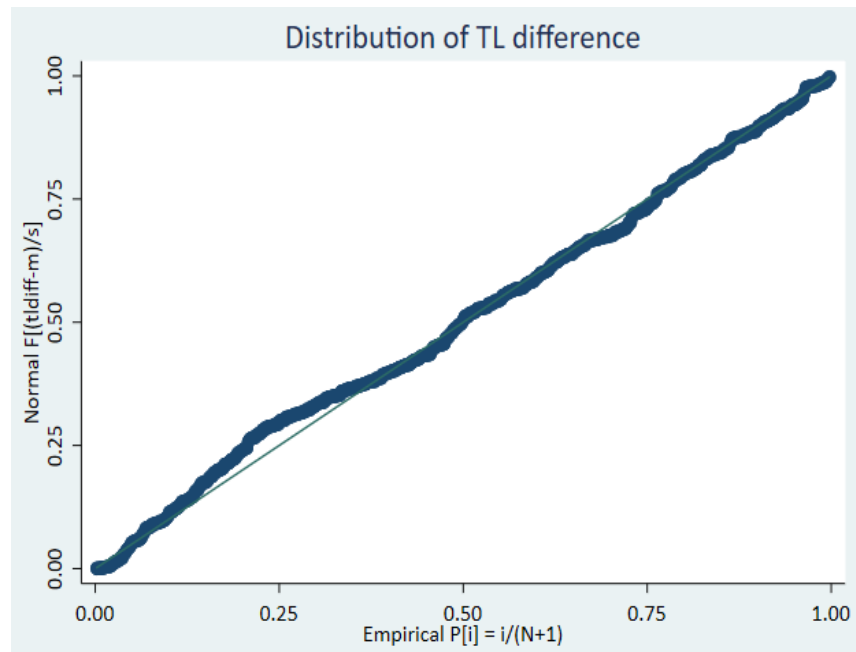

Supplement: Supplementary file 1 [file DataSheet_1.pdf]
